# Midwives’ Contribution to the Development of the Mothers’ Bond with Their Newborn

**DOI:** 10.3390/healthcare14050597

**Published:** 2026-02-27

**Authors:** Raymonde Gagnon, Amélie Garban, Diane St-Laurent, Carl Lacharité, Júlia Perarnau Moles

**Affiliations:** 1 Midwifery Department, Université du Québec à Trois-Rivières, 3351, Boul. des Forges, C.P. 500, Trois-Rivières, QC G9A 5H7, Canada; 2 Centre d’Études Interdisciplinaires sur le Développement de l’Enfant et la Famille (CEIDEF), Université du Québec à Trois-Rivières, 3351, Boul. des Forges, C.P. 500, Trois-Rivières, QC G9A 5H7, Canada; amelie.garban@uqtr.ca (A.G.); diane.st-laurent@uqtr.ca (D.S.-L.); carl.lacharite@uqtr.ca (C.L.); 3 Department of Psychology, Université du Québec à Trois-Rivières, 3600 Rue Sainte-Marguerite, Trois-Rivières, QC G9A 5H7, Canada; 4 Centre de Recherche Universitaire sur les Jeunes et les Familles (CRUJeF), Québec, QC G1C 3S2, Canada; 5 Department of Anthropology, Philosophy and Social Work (DAFITS), Universitat Rovira i Virgili, Avinguda Catalunya, 35, 43002 Tarragona, Spain; julia.perarnau@urv.cat

**Keywords:** maternal bonding, midwifery, pregnancy, childbirth, primiparity, mother–child relations, birth experience, maternal representations, continuity of patient care

## Abstract

**Background:** The mother’s bond with her newborn is important for the child’s development and their relationship. Midwives are well placed to witness first-hand the beginning of this relationship. **Objectives**: This study examined, based on mothers’ perceptions, the contribution of midwives to the development of the bond with their baby from pregnancy to the first postnatal months. **Methods**: We conducted a descriptive qualitative interpretative study in Quebec, Canada (from 2022 to 2025), with 10 primiparous mothers who were cared for by midwives in a model of continuity of care, and gave birth in a birth center, at home, or in a hospital. Semi-structured retrospective interviews were conducted between two and four months after childbirth, and were complemented by interviews with two midwives. **Results**: Most participants developed a bond with their baby during pregnancy. They discussed their midwifery care and what they felt were significant elements in the development of their bond with the baby. Midwives encouraged them to develop this bond through their approach and various means: letting them feel the fetus during palpation, talking to it, encouraging mothers to do the same, and reinforcing the bond throughout pregnancy. The birth and first moments after birth were also key moments for promoting contact between mother and baby. Midwives were also creative in promoting bonding in more difficult situations, such as when a transfer to the hospital for delivery was needed. **Conclusions**: Midwives play an important role in initiating and developing the mother–child bond during pregnancy, especially if they practice within a model of relational continuity.

## 1. Introduction

Midwifery practice is increasingly acknowledged as an essential contribution to maternity care. In 2014, The Lancet published a special issue on midwifery, emphasizing the pivotal role of midwifery services in addressing the needs of women of reproductive age, their infants, and families across all countries [1]. Renfrew’s framework for Quality Maternal and Newborn Care (QMNC) proposed focusing care on the needs of the mother and newborn with appropriate attitudes and behaviors and reducing the fragmentation of care services that focuses on identifying and treating pathologies [2]. In line with these efforts, the World Health Organization and the International Confederation of Midwives recently issued guidelines and strategies to support the transition toward midwifery care models to improve obstetric care and optimize health outcomes [3,4]. Different models of midwifery practice exist around the world, both in terms of their definition [5] and their organizational structures, where care may be shared with other professionals or where the antenatal, perinatal, and postnatal continuity may or may not be present [6].

In Quebec (Canada), the midwifery profession was only officially recognized in 1999, following repeated requests from groups of women who wished to reclaim control over their pregnancy and childbirth experiences, as well as pressure from various civil society movements [7]. This practice, which had disappeared since the end of the 19th century when medicine took over the field of obstetrics [8], re-emerged in the 1970s in response to demands from women and couples seeking a different approach [9]. It was shaped and developed based on the needs expressed by these individuals.

Midwives in Quebec work within a continuity-of-care model, meaning they provide complete maternity care in a context of normality—from pregnancy through six weeks postpartum for both mother and newborn, including the birth itself [10]. Care is provided either by a primary midwife supported by a colleague or by a small team of midwives. Birth in the context of midwifery care takes place at the location chosen by the parents: a birth center (73%), home (19%), or hospital (8%) [11,12]. Midwives attend approximately 5% of births in Quebec, as their numbers remain limited due to the late legalization of the profession in 1999 and delays in its development. The specificity of midwifery practice in Quebec is mainly characterized by the relationship established with the pregnant woman or person and the significant others who accompany her [13]. Midwives are therefore both privileged witnesses to the bond that develops between parents and their babies and key actors supporting them in the early stages of parenthood.

The bond that a mother establishes with her newborn, referred to as bonding, is a key element in the development of their relationship. Unlike attachment, which refers to the emotional connection the child forms toward the mother, bonding is recognized as an emotional state experienced by the mother toward her child, predisposing her to engage with and care for the infant [14,15,16]. This process can begin during pregnancy and continues to develop throughout the first months of the child’s life [17,18]. The way it is established will influence their early moments together, and although the bond can develop afterwards, birth and the moments surrounding it remain special times to mark this transition.

In a study we conducted on mothers’ perceptions of their first contact with their newborn in different healthcare settings in Quebec (birth with a medical-nursing team at the hospital with or without an epidural, and natural birth with a midwife team at a birth center, at home, or at the hospital) and the emotional bond they had developed with their babies, we observed differences, particularly a delay in the creation of this bond during pregnancy and around birth [19]. Most women who had been cared for by midwives reported feeling a connection with their baby during pregnancy or at the latest at birth, which was earlier than what other participants followed in other care contexts reported.

Numerous studies have examined the role of midwives across different cultural contexts and highlighted the significance of their practice [1,3,4]. In the case of Quebec, historical research has also documented the development of the profession and its particular relation with civil society [7,9]. Additionally, a different line of research has focused on the development of the mother’s bond toward her infant [14,15,16].

Midwives play an important role in supporting the development of the mother–child relationship [20]. Their observations on early bonding and on the circumstances surrounding birth, as well as their professional interventions, provide unique insights into this process. This study is the first to examine how midwives contribute to fostering mother–infant bonding within the Quebec context.

The main question of this study is: How can the different perspectives of new mothers and midwives shed light on the way midwives can contribute to the bonding process?

To answer this question, we explored how mothers accompanied by midwives perceive their first contact with their newborn and the relationship they develop with the baby in this care context. Based on interviews with mothers and midwives, we sought to highlight how midwives can contribute to the development of the relationship between the mother and her child, with the aim of identifying promising and inspiring practices for health care professionals and better addressing the needs of mothers and their babies.

## 2. Materials and Methods

### 2.1. Study Design

The methodological approach used was Thorne’s Interpretive Description [21]. This is an inductive analytical approach designed to create ways of understanding phenomena that may have implications for practice or interventions with individuals. It generates knowledge based on field data by recognizing the constructed and contextual nature of human experience [21]. It also involves practitioners as key informants to better consider contextual elements, which allows for a richer interpretation and triangulation of data.

We conducted individual interviews with ten primiparous persons who identified as women and were followed by midwives in Quebec (Canada) within a continuity-of-care model, in order to explore their perceptions regarding their initial contacts with their newborn. The sample size was not predetermined and was established during the course of the research according to the principle of saturation [22]. The number of 10 participants (women receiving midwifery care) proved sufficient, as the analysis of interviews 9 and 10 did not yield any new information. In accordance with Thorne’s approach, we also conducted individual interviews with two midwives as key informants concerning these first contacts. According to this method, only a few participants are required once the analysis of the interviews with the mothers has been completed, as the aim is simply to gain a better understanding of the context in order to nuance the interpretations and get midwives’ perceptions of some elements mentioned by mothers. This study was conducted between 2022 and 2025.

### 2.2. Participants and Recruitment

The study was conducted in a vast administrative healthcare region in Quebec, which includes both urban (medium-size cities) and rural areas, where a single team of midwives provides midwifery care (comprehensive maternity care, including prenatal, delivery and postnatal) to the entire population of that region, which is consistent with the services offered by midwives across Quebec.

Participants gave birth in a birth center, at home, or in a hospital (they had the choice of place), with a team of midwives who provided both prenatal care and continued follow-up during the postnatal period. Inclusion criteria included having given birth between 37 and 41 weeks of gestation, being over 18 years of age, speaking French, and agreeing to participate in the study. Exclusion criteria were induction of labor, instrumental delivery, or the presence of a complication requiring transfer of care to a physician during pregnancy or childbirth. All women who contacted us and met the inclusion/exclusion criteria were interviewed until data saturation was reached.

Participants were mainly recruited through flyers distributed to women in the postnatal information envelope they received after giving birth, as well as via social media networks for new mothers. An initial contact was established with future participants to verify their eligibility for the study, inform them about the research project and the implications of their participation in order to obtain their informed consent, and then to schedule the interview.

For the midwives who participated in the study, study inclusion criteria were to have a minimum of three years’ experience and hold a regular position. An invitation was sent to their workplace. When they contacted us, we verified their eligibility and we obtained their informed consent to participate in the study.

The participating women and midwives came from the same healthcare facility. However, even though the midwives interviewed were part of the same team, they were not necessarily the ones who had provided care to the women participating in the study.

### 2.3. Data Collection

Semi-structured retrospective interviews were conducted with each woman between two and four months after the birth to examine their representations of the relationship they had developed with their baby (see Appendix A.1). The interviews, lasting 60 to 90 min, carried out by the principal researcher and a research assistant, took place between July 2022 and January 2023. All interviews were conducted in participants’ homes, except for one participant who was interviewed via Zoom. Sociodemographic data were collected through an online questionnaire, along with authorization to access their birth records.

Interviews with the midwives aimed at gathering their observations of the first mother–baby contacts as well as their perceptions of their role and the factors that could facilitate or interfere with the establishment of these initial interactions (see Appendix A.2). They took place at the birth center where they practiced in April and June 2024. During these interviews, we also asked them to comment on more specific aspects that had emerged from the analysis of the interviews with the mothers (e.g., whether they had observed any signs of surprise from the mother on her first contact with her baby at birth).

### 2.4. Ethical Considerations

Prior to the interview, both the women and the midwives received the research information and consent form. Participation was voluntary, and they were free to withdraw at any time.

Ethical certifications were obtained from the university’s research ethics committee to which the researchers are affiliated and from the health center where the women gave birth (ethical certificate numbers: CER-22-284-10.02 and CER-2022-571).

### 2.5. Data Analysis

The interviews were recorded with the participants’ consent and transcribed. The analysis was conducted sequentially. First, we performed an inductive analysis of the mothers’ transcripts, which were coded and classified using N’Vivo 14 software. We first divided the transcriptions into units of meaning and assigned codes to each of them. These codes were then grouped into thematic categories to form coherent sets, allowing central themes to emerge [23,24] (see Table 1).

We also added analytical notes while referring to field notes and birth records. These steps were carried out simultaneously by a research assistant and the principal investigator, who continuously compared the emerging codes, categories, and themes. The results were then discussed reflexively with the two other principal researchers to resolve discrepancies and validate the data analysis. Emerging themes were submitted to an ongoing comparative analysis, leading to an in-depth understanding of the similarities and differences in the participants’ experiences. The themes identified within the mothers’ subjective perceptions then served as an analytical framework for the comments of the midwives, while remaining attentive to emerging elements. This made it possible to generate an interpretive description that sheds light on the mothers’ representations and experiences in relation to midwifery practice, the role played by midwives, their observations and perceptions, as well as the conditions that favor or interfere with early mother–baby contact. The analytical framework is based on the bonding process.

## 3. Results

### 3.1. Participants Characteristics

The ten primiparous women interviewed were between 29 and 37 years old (average age 33) and all lived with a partner. Four had completed secondary-level education, and six had pursued post-secondary studies. More than half were employed in the administration or education sectors, which reflects the sectoral distribution of employed women in this region [25]. Six participants had a family income below the median income for couples in Quebec. The sociodemographic characteristics of the participants are shown in Table 2.

The duration of their pregnancies ranged from 38.6 to 41.4 weeks, and their labor lasted between 3.77 and 12.65 h. Seven of them gave birth in a birth center, two at home, and one in a hospital. All of them breastfed at birth. Information regarding participants’ pregnancy and childbirth is provided in Table 3.

The two midwives who agreed to participate in our study had 10 and 18 years of professional experience, respectively. Both were practicing at the same birth center where participating mothers received midwifery care.

### 3.2. Choosing Midwifery Care

Several participants chose midwifery care because they were seeking a professional who shared their values, took the necessary time, answered their questions, and allowed them to make choices.

*I found that more humane. […] So, I decided to go with midwives for all that—the humane side, the fact that they have experience to support me when I’m not going to take the epidural, because for them it’s familiar territory.*

P29

The choice of a natural birth for the well-being of their baby was also mentioned by P28 and P24, the latter saying she disliked the fact that the epidural also affects her baby and impacts breastfeeding.

Being cared for and supported during childbirth by the same person or persons also played a role in this choice.

*I wasn’t necessarily attached to the idea of giving birth without an epidural […] but I loved the idea of being attended by someone, always the same person, who would be there on the big day. That’s it. And then when we got to know her, I’m someone who has good self-confidence, I’m in very good physical shape, so I thought: ‘There’s no reason why it shouldn’t work without an epidural.’*

P12

The place of birth also influenced the choice of some participants, including P28:

*When we decided to try to have a child, it was immediately clear that I would give birth there [birth center] because I thought it was beautiful […] and there was no way I was going to the hospital. I want it to be a family affair, a human experience. I don’t want it to be in a room with my legs in stirrups.*

P28

**Figure 1:**
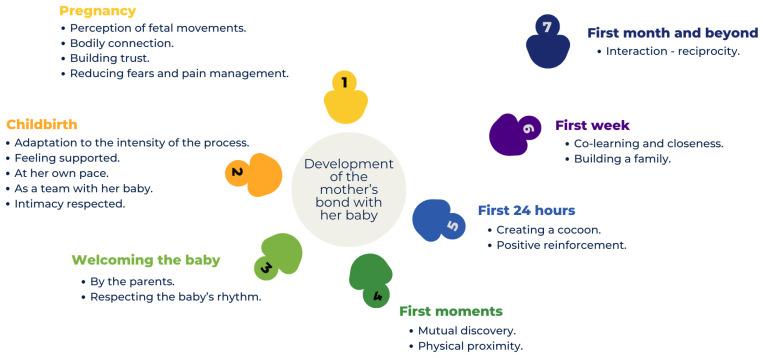
Mothers’ experience regarding the development of the bond with their baby in the context of midwifery care.

Mothers’ experience in creating a bond with their baby will now be examined from their perspective, and we will use midwives’ perceptions as a way to complement mothers’ perspectives in order to better understand how midwives intervene in the initiation of the mother–child relationship. Figure 1 illustrates the development of the mothers’ bond with their baby across the different stages from the pregnancy to the first months after birth.

### 3.3. A Bond with the Baby During Pregnancy

Several participants established a bond with their baby during pregnancy. For P20, this bond was tangible from the very beginning: ‘*The moment I saw “pregnant.” It was just a magical moment, that too. […].*’ She then developed a relationship of trust with her baby: ‘*Throughout the pregnancy […] I trust my baby and my body*.’

The baby’s movements became a reassuring presence for P12. For P26, being attentive and listening to her baby’s movements made her feel connected ‘*It was a really beautiful pregnancy. I felt connected to him. And you know, I talked to him sometimes*.’ She added that something happens in the mother’s womb with the baby, because he recognizes the sounds he has heard: ‘*It’s as if he recognizes the voice. He becomes very attentive when they [midwives] talk*.’

Midwives supported women throughout their pregnancy. During follow-up visits, they let parents listen to the fetal heartbeat, and during palpation they talked to the baby and encouraged the parents to do the same. This led P28 to say that:

*The feeling grew stronger with each appointment with the midwives because they make it very human. You hear the heartbeat every time, they touch your belly and make you feel that there really is a baby. So, I think that at every appointment I felt something growing inside me.*

Women felt reassured by midwives about pain management, which strengthened their confidence and prepared them to be more receptive to the bonding process in order to welcome their baby.

*I told my midwife many times that I was afraid of the pain, that I didn’t know what to expect, that I was scared I wouldn’t be able to do it and wouldn’t have the courage to go all the way. And she reassured me by saying: ‘You don’t need to worry because when your body starts naturally, it’s little by little. Then at some point, it gets stronger and stronger, but your body has adapted and gotten used to it. So, you can do it. Your body can do it.’ So, she really encouraged me a lot.*

P28

Thus, they encouraged women to use various ‘active’ methods of birth preparation and pain management: reading, exercises to stay fit, yoga to work on breathing, meditation that encourages the mother to talk to her baby (P28), raspberry leaf tea to prepare the uterus, perineal exercises (P8), and they also discussed positions for pain relief.

*So, about the pain—you know, for me the epidural wasn’t even an option. That’s it. I had told my midwife: ‘Can you just find me some positions where it will go as quickly as possible, in the sense that if I get stuck at any point, you know, get me moving.’*

P29

Several mentioned adopting a confident attitude during pregnancy and childbirth: ‘*I felt really confident where I was with the people around me*.’ P12

### 3.4. Childbirth: A Powerful Moment

The participants provided many details about their childbirth experience. They talked about mentally preparing themselves not to have an epidural and feeling confident in the normality of the process, which, according to them, the baby also feels. Through their attitude and actions, midwives created conditions that facilitated the mothers’ bond with their baby.

First, midwives consider that the mother gives birth to her child; they do not deliver the baby. Thus, P12 and P11 considered that they did it as a team with the baby and their partner. The midwife was there to support them, but she did not deliver the baby. All of this contributed to the development of the bond with the baby:

*Precisely the fact that we did it together. The fact that he was born with his dad, with me, was like we were a team. It wasn’t… Sometimes I read books and it’s like, oh, the midwife delivered my baby. For me, the midwife was there, she really helped me, but we did it together […]. So this bond, it really is… it’s, it’s in everything we do.*

P11

In addition, midwives were both present and respectful of the parents’ intimacy. For instance, P20 felt as though she was in her own little world, alone with her baby and partner, as she was aware of the midwives’ presence but not what they were doing.

*I find it magical because you’re really in your own little world. You know, at one point, there was more than one midwife, but I didn’t know that. Yet, they were around me, talking to each other, touching me, but I never noticed.*

P20

And they respected the parents’ choices: ‘*It was our choice to do it that way, and they respected it as much as they could.*’ P28

During labor, in general, participants described feeling a sense of flow that was adapted to their own rhythm. Midwives encouraged speaking to the baby: they talked to the baby and invited parents to do the same, which fosters a bond with the baby. They also respected the mother’s pace without putting pressure on her.

*I didn’t want to have that pressure behind me from someone who wants to give me an epidural, or who wants things to go faster and for me to give birth in two hours. I didn’t want any pressure. […] I wanted it to be gentle. That was a big part of choosing midwifery care.*

P26

Midwives ensured that labor and birth went well and encouraged women throughout the birthing process.

*And then at one point, they listened to the heartbeat. You know, I’m sure they do that all the time, but you know, it’s like, okay, his heart is doing well. You know, my midwife was there all along, saying: ‘Okay, that’s good, that’s fine! You’re doing great! It’s going well! Keep going!’*

P20

During the pushing phase, several participants were very focused on themselves and what they were feeling—such as fatigue, pain, or because the birth seemed to be progressing too quickly. In contrast, others described a strong bond with their baby. For example, P11 spoke about the connection she felt with her baby during pushing: ‘*Yeah, well, childbirth is like, really, that’s when you really connect*.’ P27 even mentioned empathy for her baby, explaining that natural birth is beneficial for her baby and that he can feel it. P26 mentioned that the birth conditions allowed her to develop a bond that was both ‘*strong and gentle.*’

As mothers explained, midwives created favorable conditions for the mother to be ready to create a bond with her baby—by fostering confidence, encouraging acceptance of contractions to dispel fears, or suggesting visualization, talking to the baby, and even using metaphors.

*When it was time to push… the midwife was stroking me and telling me to visualize my baby coming out into a nest of feathers. She kept saying: ‘Think about your nest of feathers. She’s coming. It’s going to be smooth and gentle.’ She talked to me a lot. So, I talked to my baby too at that moment, because she was whispering in my ear: ‘Focus, you’re going to bring her out, she’ll be in a bed of feathers, she’ll be fine.’ So that’s what I did afterwards, throughout the pushing phase. I was talking to her in my head. I was talking to her and touching my belly.*

P28

Others stayed in contact with their baby by feeling it move during descent and touching its head:

*You’re really focused on yourself and your baby. Like, where is she now? And my midwife was there with me, so at one point she said: ‘Do you want to touch her? You know, you can feel her head.’ So, I was like, yeah. I reached out and touched her head. And it was a really wow feeling. You know, like, OK, she’s coming. She’s right there. And, you know, like I said, I was really well supported.*

P20.

### 3.5. Giving Birth and Welcoming Your Baby at Your Own Pace

According to the participants, birth took place in an intimate setting, at the pace of the mother and baby, with little outside interference. The mothers considered themselves active participants in the moment. It was both a powerful experience for the mothers and a significant moment meeting their baby, as well as a personal sense of empowerment and pride. One participant mentioned feeling self-confident, strengthened by the serenity provided by the birth conditions, which allowed her to connect with her baby.

*That was my wish to give birth at the birth center, to have that bubble. And it was really respected. I had the intimacy I wanted, that little bubble. I felt completely comfortable speaking as loudly as I wanted, being naked, being… I felt really free to be myself and take as much time as I wanted.*

P8

At birth, midwives encouraged parents to welcome their baby themselves. P26 and P28 mentioned that being the first (partner or mother) to hold their baby was very important in creating a bond.

*It was my partner who took the baby, actually, to bring him out. Then he placed him on me. So that was really a beautiful moment. I remember that precise moment—my partner crying and holding our child in his arms… You know, he was the one who had the first contact. So, it was truly a privilege. And I think it made the attachment bond with my baby even stronger because it wasn’t the midwives who touched him, you know, it happened between the two of us.*

P28

Midwives continued to respect the parents’ pace by offering them to hold the baby when they were ready (one mother needed to touch her baby before taking him in her arms). In addition, physical contact with the baby appeared to be highly significant for most participants. At birth, the connection was established through touching and hearing the baby, and the feeling of having worked together with the baby. These conditions created an atmosphere where feelings of love could emerge and spread.

One midwife interviewed mentioned that:

*The smell and warmth of a baby are soothing for the mother. It also helps promote breastfeeding. And we know that breastfeeding releases oxytocin, which is a hormone associated with love and attachment. So all of this fills us with endorphins. The whole cascade of hormones that is important.*

RM1

All of this helped to create an atmosphere of well-being, promoting parent–baby bonding and interaction. Midwives also emphasized the importance of allowing the baby to feed at their own pace, without interruption:

*I saw such a difference on breastfeeding initiation and mother-baby contact that I really changed the way I do things. I systematically wait until the baby has finished feeding. If it takes him/her two hours, that’s fine, then it will take him two hours.*

RM2

In summary, midwives created conditions that facilitate the development of the bond between mother and child:

*I truly believe that birth conditions really influence the confidence you have when the baby arrives. That’s for sure. But that’s for sure it made things easier because everything went well, so we stayed with a positive experience. There was no fear, so I wasn’t afraid, and so she mustn’t have been afraid either, because she was in great shape and so was I. (…) So, it allowed me to feel confident, and she surely felt that I was confident.*

P12

An “indescribable and wonderful” moment was reflected in the emotions mothers expressed: joy and pride associated with a sense of accomplishment in giving birth, and the deep feeling of love they experienced, either instantly or gradually. Several mothers described the continuity of the bond established during pregnancy with their baby, which continued after birth, notably through the recognition after birth of the baby’s movements they had perceived during pregnancy.

Some participants mentioned that the process unfolded naturally and normally, whereas, on the contrary, another woman had not anticipated what would happen after the birth.

### 3.6. Evolution of the Bond During the First Moments After Birth

Many mothers expressed pride and a deep sense of admiration for their baby, enjoying a moment of well-being in symbiosis. They felt that the emotional bond continued to evolve during the first moments after childbirth. It could be a gradual love, a sense of belonging and possession (“this is my baby”), or a feeling of connection.

In addition, half of the participants mentioned the need to get to know their baby, to get acquainted or to meet each other: ‘*But the first half hour was that, mainly talking to him: I wanted to explain all about life to the baby and tell him…*’ P8.

Midwives recognized the crucial importance of the physical and emotional bond between mother and child during the first hours after birth. They emphasized rhythm, closeness (parents are in the same bed as the newborn), and the intimacy of parents with their baby, which contributed to the development of the bond between the mother and her child.

Midwives observed a strong need in mothers for physical closeness with their babies:

*They need to be close to their baby who was in their belly. I think that makes sense. There’s like a need to watch over the baby. It’s probably an instinct because all women are like that. They’re kind of on a high. Yet, they’re supposed to be really tired, but they don’t sleep; they need to observe everything the baby does, at least during the first 24 h.*

RM2

Skin-to-skin practices and immediate contact were considered important for promoting breastfeeding and the development of the bond, as P12 said: ‘*We just had to stay close and admire him. We didn’t need to think*.’ (P12).

According to the mothers, midwives always tried to prioritize the contact between the parents and the baby, for example, before checking for maternal tears, or when assisting the baby immediately after birth, or when a transfer was deemed necessary. They also examined the baby with the parents, explaining the baby’s behavior and what they were doing. Midwives took care of the baby, but also the mother, ensuring her comfort, which facilitated the transition to the postnatal period.

It should be noted that certain elements could interfere with the contact with the baby, such as the umbilical cord pulling while waiting for the placental delivery or, as one mother mentioned, having to leave her baby for a few minutes to take a bath. Another mother felt unsettled when changing her baby’s diaper, even though she had done it for other babies before. Some mothers may also have experienced difficulties with initiating breastfeeding.

*The first few days, breastfeeding didn’t start the way I wanted. So, of course, it was really difficult. […] But the midwives supported us. They were incredible. Because you really need to be determined if you want to breastfeed.*

P24

Midwives mentioned the importance of support and available resources, emphasizing that successful breastfeeding depends not only on the mother’s commitment, but also on the support she receives. It is interesting to recognize the interdependence between the experiences of childbirth and breastfeeding.

Indeed, mothers mentioned that breastfeeding helped strengthen the bond with their baby: ‘*As soon as she takes it [the breast], she is immediately comforted. You know that it’s like such a beautiful bond’* (P28). According to P12, it is a special moment: *Well, when I was breastfeeding her at night, I would think: ‘Ah, this is our moment together. It’s just the two of us. I’m the only one who can do this.’ I thought it was great.*

### 3.7. The First 24 h: The Beginning of Family Life

During the first 24 h, if the birth took place in a hospital with a midwife, she helped the parents get ready to go home (e.g., changing the baby’s diaper, helping the mother get dressed). In a birth center, the birth assistant was present to ensure that the baby was breastfeeding properly, reassure the parents about returning home, and explain the administrative procedures.

Mothers who gave birth at home said they felt well supported and confident. Regardless of the place of birth, midwives continued to provide support by visiting them during their first days at home. They provided assistance, particularly with breastfeeding, and offered positive reinforcement, which according to most of the mothers we spoke to helped strengthen their confidence. However, two participants, P11 and P28, mentioned anxiety and stress related to the responsibility of having a child. They wondered whether they were “doing it right” or if their baby was “okay”.

The need to be alone and close to their baby and partner fostered the creation of the bond with the baby. Some participants mentioned that being in a cocoon-like environment helped them become a family. During this period, the mother’s bond with her baby developed through contact, including eye contact, as well as verbal and nonverbal communication, and her interpretation of what the baby was feeling.

One midwife (RM1) highlighted the benefits of giving birth in a less medicalized setting, such as a birth center or at home, in terms of parents’ well-being and bonding with their baby. She explained that a mother who gives birth in these environments can benefit from an early leave, often just a few hours after birth, without the intensive monitoring found in hospitals. This allows the mother to stay in sync with her baby’s natural rhythm.

She explained that, by respecting the baby’s sleep and feeding cycles, parents are not forced to wake him or her every three hours for feeding. This enables them to rest more and feel in better shape, which is crucial for their ability to care for their child. The midwife interviewed emphasized that well-rested parents are more likely to interact positively with their baby, thereby fostering a stronger bond with him or her. Conversely, exhausted parents may find it difficult to meet their child’s needs, which can negatively affect the quality of their interaction and attachment to the baby.

It is worth noting that one of the midwives mentioned that some women who had epidural births told her about the difference they felt after experiencing a natural birth:

*It made me realize… and the women, they explained to me that they had experienced several births with an epidural in the hospital. And they told me that (with midwifery care, without epidural) they felt an immediate connection with their babies. Before, they had more difficulty taking care of their baby from the start, in the first days. Sometimes they told me that ‘when you were in a hospital setting with an epidural, it took a little longer to establish that strong bond.’*

RM1

She added that women who had been transferred for medical reasons also faced more difficulties in the first few days, likely due to physical fatigue, but also having to mourn the desired birth experience.

### 3.8. A Co-Learning Experience for Mothers and Babies During the First Weeks

During the first weeks following birth, we note that the bond with the baby evolved. P28 mentioned a gradual love, whereas P11 and P8 described a very strong attachment bond present from the earliest weeks.

In addition, P8, P12, P20, and P26 also mentioned that developing reference points is a day-to-day learning process that takes time and involves trial and error. P12 described it as a co-learning process, where she and her baby got to know each other and build mutual trust:

*When I put her to bed, I remember clearly, she cried, I held her again and said: ‘No, look, I need to sleep and so do you. I’m going to lay you down on your belly.’ I kept my hand on her and I saw that she was really calm, that she was falling back asleep. I said: ‘It’s OK, everything’s fine! Perfect.’ We talk to her a lot like a normal adult. I think that helped too. I told myself: ‘She’s sleeping well, she seems to have understood what I said. That means she has trust, she trusts us too.’*

P12

During the first weeks, for most participants, the bond between mother and child developed and manifested itself through interactions and reciprocity experienced in different ways, such as the ability to respond to the baby’s needs or communicate with him or her, the baby’s expressions and smiles: ‘*After the birth, until she smiled, it was more of a contact relationship that I had with her and that I could enjoy her*. (P29)’. Also, the fact that the mother made the baby talk or talked to the baby: ‘*I had trouble breastfeeding him […] but we talked together all that time. We told ourselves, we were going to make it together (chuckles). We talked a lot together.* (P11).’ Some mothers “provoked” reciprocity by talking to their baby, which helped to create and strengthen the bond that grew over time.

Physical contact, intimacy, and closeness also fostered the development of this bond. P11, P20, P26, and P28 felt the need to be constantly in close bodily contact with their babies, sometimes in an almost instinctive, animal-like way—whether during sleep, through skin-to-skin contact, or simply by touching the baby’s hand.

A midwife observed that it is often when parents return home that they truly begin to feel like a family—when the environment becomes more comfortable and they have to rely on themselves. She described the transition of new parents from the hospital to their home and the impact of this transition on their confidence and autonomy as parents. She emphasized that, once at home, parents feel more at ease and gain confidence.

In the hospital, parents are often taken care of and tend to rely on professionals for advice about their baby. They ask questions and expect immediate answers from the medical team. In contrast, once at home, ‘*it’s just them—they have to rely on themselves. It’s like they spread their wings as parents.*’ (RM2)

The midwives pointed out that when childbirth did not go as the mother had planned, it could be difficult for her to bond with her baby. However, this bond could be strengthened through positive experiences during postnatal care. For example, a mother who expressed discomfort in connecting with her child was offered a “birth refacilitation” [27]. This approach consists of recreating an environment similar to that of childbirth to release the emotions experienced during birth. This type of intervention illustrates the importance given to postnatal follow-up in developing the mother’s attachment bond. Midwives observed that, around two weeks after birth, mothers often went through a period of mourning related to their expectations of childbirth. Once this period was over, the relationship between the mother and her baby tended to strengthen.

### 3.9. During the First Few Months: A Reciprocal Relationship Where Balance Is Established

During the first months following birth, mothers said they were reassured about their concerns and received answers to their questions. They may receive support and advice on how to bond with their baby.

Participants seemed to have found a balance between respecting their baby’s rhythm and needs and their own needs. They took time for themselves and had developed a routine. The relationship was reciprocal, as the baby was seen as ‘a human being who reacts.’

*Now, I sort of got to know her. I’m learning to adapt to her rhythm of life, but it’s going really well. I do it with respect. I fully respect her. You know, like, well, she’s not a baby who sleeps a lot. So, it’s like, well, you don’t sleep, so we go to the playground. We have fun. I just go with the flow. I’m not stressed with her anymore […] For her second month of life, it feels like I’m super relaxed with her. I let things happen and I have no expectations of her or of myself. It’s just as it comes, and I’m happy that it’s going this way.*

P28

Finally, one of the midwives pointed out that there were several ways to bond with your child:

*It can happen instantly, or it can happen after six months or a year. Sometimes you need to give it time and be patient. I try to ask questions, encourage them to express themselves, not feel guilty if it’s not… Often it comes out later. You know, women tell me afterwards: ‘You know, I didn’t fall in love with my baby right away like I thought I would.’ So, we try to normalize it and say: ‘There are plenty of women like that, but no one talks about it because everyone feels ashamed.’*

RM2

In light of these results, five themes emerged regarding the creation of the mothers’ bond with their baby in relation to the support provided by Quebec midwives: felt experience, rhythm, confidence and associated consequences, environment, and continuity. The main themes characterizing midwife support and its influence on bonding are shown in Table 4.

## 4. Discussion

The choice of care during pregnancy is decisive for the subsequent experience of labor and birth. Women are often not informed about the different types of childbirth, and yet, choosing a type of care leads them towards a specific approach to preparation and childbirth, shaped by a particular conception of birth [28]. Furthermore, pregnancy and childbirth are social processes embedded within the context of social norms and expectations specific to the environment. The social construction of these events influences the behaviors of pregnant women [29]. In Quebec society, the choice to be cared for by a midwife remains marginal, as the majority of women are followed by a physician or an obstetrician–gynecologist.

Based on the results of our study, it appears that entering the process of care with a midwife contributed to the early establishment of a bond from the mother to her baby during pregnancy, which grew over time. Mothers seemed to be connected to their babies, and the support provided by midwives facilitated and encouraged this bond. Analysis of the interviews revealed five themes related to the creation of the mothers’ bond with their baby, highlighting different aspects of midwifery practice that seemed to contribute to the establishment of this bond. These included the mothers’ receptiveness and ability to feel their baby throughout the process, the midwives’ respect for mother–baby rhythms, the importance of strengthening the mothers’ confidence and the environment in which they evolved, as well as the continuity of care offered by midwives from pregnancy through the first months of the child’s life (see Table 4). These various elements, which seemed to have been determining factors in the creation of the bond between mothers and their baby, are detailed below.

### 4.1. Encouraging the Felt Experience

In our analysis, felt experience emerged as a key factor in the creation of the bond between the mother and her baby. Felt experience can be defined as the physical, psychological, sensory, and emotional sensations experienced by the mother. It is a form of subjective consciousness that links sensations to emotions [30]. Results of the present study revealed that the way midwives interact with parents and structure the environment (calm, intimate, familiar) creates favorable conditions for mothers to fully live the “felt experience” at all stages. Rather than focusing primarily on monitoring and care—although they remain vigilant and intervene when necessary—midwives prioritize contact with the baby starting during pregnancy. They invite mothers to turn inward, talk to their baby, as well as touch and welcome their baby. They foster intimacy, closeness, and calmness.

Several authors [31,32] have explored the effects of mindfulness approaches and body–mind centering combining movement and touch [33] in order to focus on the “soma” during and after pregnancy to strengthen the mother’s emotional bond with her child as well as the attachment and quality of the parent–child bond. Felt experience seems to play an important role in rendering more concrete the existence of their baby for mothers during pregnancy, but also in raising awareness of the birth, which reinforces the bond between mothers and their baby [34]. The mothers’ bond toward their baby develops during the prenatal period through interoception (maternal focus on bodily sensations), perception of fetal movements [35] and communication with the baby [36], as well as through a state of ‘reverie,’ understood as a psychic capacity to welcome what the baby makes the mother feel and to project his or her existence [37].

In addition, when midwives encourage pre- and post-natal body techniques (haptonomy, breastfeeding, skin-to-skin contact) [38] and close mothering at birth (touching, caressing, providing care), they contribute to the transition from the inner to the outer world acting like a “bridge between the two phases,” for the mother and baby [39]. Establishing an emotional bond between the mother and her child, as well as contact, also promotes the initiation of breastfeeding, which in turn strengthens the mother–child bond [14,40,41].

### 4.2. Respecting the Mother–Baby Rhythms

The present study highlights the importance of respecting the rhythms surrounding birth, as this allows mothers to integrate their experience and positively promotes the establishment of the relationship with their baby. Indeed, when time slows down, the birth environment becomes a place of “connection and revelation of new bonds,” allowing the “apprentice mother” to become aware of her new role [42].

We also observed that the feeling of having enough time is fostered by midwives’ practice from the very beginning of their relationship with women during pregnancy. Study participants said they appreciated that their midwife took the time to actively listen to them and respond to their concerns. Respecting the mother’s rhythm also seemed to contribute to ‘maternal reverie’ [43].

As reported by study participants, the relational dynamic continues during childbirth, where the idea of collaborative work between the mother and the baby, as well as the importance of adapting to each mother’s rhythm are promoted, which seems favorable to the development of the mother’s emotional bond with her child. At this stage, mothers seek professionals who do not pressure them with interventions aimed at speeding up the process, but rather reinforce the feeling of having all the time they need. In this regard, midwives have tools and criteria for assessing the progress of labor that are not based solely on time limits, allowing for greater flexibility and better adaptation to the characteristics of each birth. However, in certain settings (particularly when practicing in hospitals), midwives may feel compelled to comply with obstetric timeframes, which can lead them to perform interventions they would not otherwise carry out in a different context [44].

In the postpartum period as well, our study showed that time and respect for each family’s rhythm are essential to creating the mother–child bond. Thus, the context of the first contact appears to have fostered the development of the bond with the baby among study participants. They emphasized the importance of spending intimate, quality time with their baby and partner, being able to actively initiate breastfeeding with appropriate support if needed, and enjoying skin-to-skin contact and closeness in general.

Furthermore, as mentioned by one of the midwives interviewed, it is important to consider each person’s individuality and wait until mothers are ready for physical contact and closeness. It has been shown that when this contact and physical closeness occurs at the time desired by mothers, they are less likely to have difficulty establishing an emotional bond with their baby [38]. In addition, Stoodley et al. [40] reported that the first night after birth is a special time for many mothers, as they can spend time alone with their baby, allowing them to bond with him or her.

However, they pointed out that in the immediate postpartum period in hospitals, the time and space needed to establish a strong emotional bond between the mothers and their baby are sometimes not prioritized [40]. Women who did have access to these elements, along with participating in decision-making and feeling supported, experienced a more positive birth and were more willing to care for their newborn [45]. In short, it is about putting aside clock time to make room for each woman’s subjective time —a time in which the physiological, emotional, social, and spiritual transcendence of birth can be acknowledged, a time ‘to savour, appreciate and ingest the profundity of meaning around childbirth’ [46], p. 141.

### 4.3. Developing Mothers’ Confidence

It is important to remember that pregnancy, childbirth, and the postpartum period are new experiences for first-time mothers and their families, which generate numerous uncertainties and anxieties, as expressed by our participants and also reported in other studies [20]. It is therefore important for healthcare professionals to strength mothers’ confidence. In our study, we observed that midwives did this in different ways.

First, the care they provided focused on normality rather than on risks, promoting birth as a normal and positive experience. They provided emotional support to new mothers during the perinatal period, which they reported as being important for strengthening mothers’ confidence and autonomy, thus influencing the development of the mother’s bond with the child.

Several participants also mentioned that when midwives provide information about the physiological process and the smooth progress of their pregnancy, it reassures them and builds their confidence in themselves and their bodies. Thus, the midwives who provided care to the women in our study demonstrated active listening, which fostered women’s trust in themselves, their own bodies, and in their babies. Studies have shown that the midwives’ active listening [47], along with the emotional and physical support offered to new mothers during the perinatal period [39], provide a sense of security and reduce fears and uncertainties [48]. Strengthening confidence in mothers’ physical [36] and emotional abilities [49] allows them to open up to perceiving sensations and bonding with their baby during pregnancy, which further reassures them and helps them recognize themselves as mothers [20]. As noted by Dubber et al. [50], parents attribute characteristics to their baby during pregnancy, partly based on perceptible fetal reactions. In our study, midwives drew mothers’ attention to their babies during pregnancy. Participants reported that being attentive to their baby’s movements during this period helped them establish a connection with the baby in the early moments after birth, as they recognized the same behaviors they had observed in utero. Furthermore, the mothers we interviewed expressed pride in successfully caring for their baby, which strengthened their sense of agency and confidence [43]. It is interesting to note that, when mothers feel more confident, they are more likely to provide adequate care [20,51]. It is therefore important for health professionals to promote a sense of confidence in mothers, which will help create the mother–child bond, as demonstrated by research conducted in different cultural contexts [20,40,51].

In sum, mothers’ self-confidence contributes to fostering the creation of the bond with their child. Although it is a personal attribute, our study showed that this feeling of self-confidence as a future mother can also develop through the relationship with midwives. Indeed, a trusting relationship with midwives increases the overall sense of satisfaction, improving women’s comfort and allowing them to feel free to be themselves, as explained by one of the participants we interviewed, and to act according to their instincts, which positively influences the relationship with their baby [40]. Furthermore, the trust between midwives and mothers, built progressively over time, enables women to feel safer, less stressed, and more respected [6]. It also increases overall satisfaction [47,48]. This is why the relationship that women establish with healthcare professionals, and more specifically with their primary care provider, will be decisive in managing their emotions and will influence how they experience the birth.

Finally, participants mentioned that midwives promoted calm, peaceful birth conditions that prioritized mother–baby contact. For example, many appreciated being the first person to hold their newborn or enjoying quality time alone with the baby in the early moments after birth. They explained that this strengthened their confidence and helped them connect with their infants and become a family. Our results are in line with other studies that have shown that birth conditions that provide serenity [52], intimacy, and closeness with the baby reinforce mothers’ confidence and encourage them to bond with their newborn in an environment conducive to discovering the new family [40].

Thus, as reported by study participants, midwives responded and adapted to each woman’s needs by individualizing interventions to make them more relevant. They were able to do this because they have the knowledge and professional culture necessary to strengthen mothers’ self-confidence, as suggested in the literature [44,48]. Therefore, midwives must be trained and equipped to foster the relationship between mothers and their baby [40].

### 4.4. Creating a Favorable Environment

Mothers’ confidence in their parenting abilities is also fostered by the birth environment. In this sense, midwives create and maintain a care setting that aligns with their practices and conception of birth, whether in a hospital, birth center, or at home. They can create a social, physical, and organizational environment conducive to building the bond between mothers and their baby. First, they make space for the mothers’ companion, providing a reassuring and safe birth environment. Second, midwives take environmental conditions into account and adapt them to promote women’s comfort during childbirth. In fact, study participants expressed a desire to give birth naturally in order to have a calm environment and a more intimate and “humane” birth experience. For one participant, the calmness of the environment and the care provided, along with the sense of self-confidence instilled by the midwife, were key elements in creating a bond with her baby.

Research has shown that a ‘comfortable and nurturing’ environment promotes the mother–child connection [42]. Expectant mothers need to create a ‘nest’ [53,54] that is both comfortable and supportive, which contributes to the establishment of their bond with their baby. More than just a functional space, this environment welcomes emotions and facilitates the transition to motherhood, regardless of the place of birth [55].

In this regard, it is more difficult to create and maintain these conditions in a hospital setting because the environment shapes midwives’ actions [44]. However, midwives can also act as guardians of the ‘Birth Territory,’ meaning they ensure that mothers can experience labor and birth without disturbance, safely, and fully experience their bodily sensations, regardless of the place of birth [56]. Beyond the physical aspects already mentioned, the role of midwives helps create a safe and trusting environment that respects each person’s natural rhythms and promotes women’s autonomy and the mother–child relationship [57].

One study [44] showed that home environments and birth centers allow midwives to be more present for women, with autonomy and without distractions. As highlighted in some studies [58], home birth offers certain beneficial characteristics for the creation of the mother–baby bond: a familiar space, surrounded by trusted people [39], intimacy, and a calm and secure environment that facilitates the process and reduces stress factors associated with hostile or unfamiliar settings [59]. In midwife-led units or birth centers, the objective is to reproduce the characteristics of the family home that support the physiological development of childbirth and the immediate postpartum period, as well as the establishment of a strong emotional bond between mothers and their baby and within the family as a whole [60].

Our study showed that, during the immediate postpartum period, being at home or returning home within the first hours after giving birth promotes closeness and contact between mothers and their baby. As observed by the midwives interviewed, this also facilitates adaptation to the baby’s sleep and feeding cycles, allowing parents to rest better. Mothers also mentioned the importance of home visits by midwives, who provide support and positive reinforcement in their new role. Furthermore, the midwives interviewed reported that returning home helps parents gain confidence and strengthens the sense of family.

Other studies have also indicated that being at home promotes uninterrupted skin-to-skin contact between parents and the baby, which is one of the main elements in building emotional bonding and initiating early breastfeeding [61]. In addition, postnatal home visits improve satisfaction, family involvement, and the development of parenting skills for both fathers and mothers [62]. By providing care to mothers, not only during pregnancy and childbirth, but also postnatally, midwives also support a positive birth experience and strengthen mothers’ emotional bond with their baby [39].

### 4.5. Providing Continuity of Care

Continuity appears as an important element expressed in different ways, but mainly, through pre-, peri-, and post-natal care. The results of our study showed that the bond developed by mothers toward their baby during pregnancy continues to be maintained or strengthened during childbirth. What was established with the midwives’ support in the prenatal period continues during childbirth and is reflected in talking to the baby, working as a team with the baby during birth, which is an interesting contribution of our study. Indeed, it had been established that interventions that are repeated during the prenatal and post-natal periods are more effective in strengthening this bond [20], but experiences during labor and birth do not seem to have been documented. Yet, women’s experiences are part of a continuum, and childbirth should not be overlooked by professionals, as it is, above all, about bringing a child into the world and not merely an obstetric procedure.

Furthermore, continuity of care with the same midwife or a small team of midwives contributes to enhancing women’s confidence. Participants’ comments reflected a sense of safety and connection established with the midwife during the prenatal period that continued into the postnatal period, as noted in other studies [63,64]. Continuity of care throughout the nine months of pregnancy allows the mother to build a stable, reliable, and reassuring relationship with the healthcare team [39]. The midwife becomes a source of support and reference throughout the process. Moreover, having a known midwife during labor also promotes a positive birth experience [65] and helps reduce maternal anxiety, attenuate pain perception, and improve the sense of control [66]. In addition, the better the midwife knows the woman, the more she can adapt to her rhythm. All these elements seem to work in synergy, enabling the mother to focus on what she is experiencing and feeling and to connect with her baby. Postnatal home visits by midwives were also considered important by mothers in the present study, as noted in the literature review by Walker et al. [67]. These postnatal visits completed the experience by supporting first-time mothers in their new role. Continuity of care during the pre-, peri- and post-natal periods is therefore beneficial [40] and contributes to the development of the bond between the mother and her child as well as the transition to family life.

### 4.6. Strengths and Limitations of the Study

This study highlights, from the mothers’ perspective, how midwives can promote maternal bonding and the development of the mother–child relationship. The mothers’ reflections regarding their experience, obtained soon after birth, proved particularly insightful as they provided a window into how the maternal bonding process unfolds from pregnancy to the first months after birth, and the specific contribution of midwifery practice to this process, particularly through relational continuity and experiential care. The mothers’ comments clearly showed the elements that contributed to the establishment of this bond. The study makes a significant contribution to understanding the role of midwives in this important aspect of early parenthood and child development, which is scarcely addressed in the literature.

The description of the theoretical framework, data collection tools used and analytical approach, as well as the nuanced presentation of participants’ experience enhance the study’s transferability, particularly in contexts where midwifery practice is similar. That being said, the detailed results on ways to promote the bond between the mother and her baby, supported by numerous participant quotations, may be useful to midwives or other health practitioners in different settings, beyond the Quebec midwifery context.

Furthermore, the measures implemented throughout the data analysis process—both through dual coding and interpretation by two researchers, and through the reflexive approach deployed during the numerous sessions of discussion, comparison, and validation with two additional researchers—provided methodological rigor to the study.

Moreover, this study examined the establishment of the bond between the mother and her infant in the context of normal births, which has rarely been documented. However, excluding medicalized births (i.e., induction or instrumental delivery) limits the generalizability of the findings, particularly to higher-risk or hospital-based populations. Additionally, the participants were recruited on a voluntary basis, which may introduce a selection bias. Other limitations of the study include potential recall and social desirability biases. Furthermore, considering that participating mothers had self-selected midwifery care over other types of healthcare surrounding pregnancy and birth, we cannot rule out a potential idealization or positive selection bias.

Another limitation of our study is that the women and midwives interviewed came from the same healthcare facility (although it did cover a vast region including both urban and rural areas) and had similar sociodemographic characteristics. However, this study was not intended to be representative of the overall population of women receiving midwifery care in Quebec, as doing so would have been too demanding and costly for a qualitative study.

In addition, the participating midwives were not necessarily those who accompanied the women interviewed, but they were part of the same team. Furthermore, since pregnancy and motherhood are socially constructed, the strategies used by midwives in Quebec to foster the mother–child bond will need to be adapted to other cultural contexts. Likewise, it is important to consider the professional context and the practice model in which midwives work in Quebec, although the examples provided may inspire midwives and other health professionals practicing in other contexts. Future research could explore the perspectives of fathers or partners on the factors that foster their bonding process with their baby, and how midwifery care may contribute.

## 5. Conclusions

Our study reported on the different perspectives of new mothers and midwives on how midwives can contribute to the maternal bonding process. Several elements of Quebec midwives’ practice contribute to mothers’ bonding with their child: encouraging pregnant women to be receptive and feel their baby throughout the process (during pregnancy, labor, and childbirth), respecting the mother–baby rhythms, strengthening the mothers’ confidence, creating a reassuring environment, and ensuring continuity of care from pregnancy through the first months of the child’s life. All these elements appear to have been determining factors in creating the mothers’ bond with their baby. These findings show that the mothers’ bond with their child starts before birth and can be encouraged from the beginning of pregnancy. The idea that childbirth is a collaborative effort between the mother and the baby, as well as talking to the baby during the process, helps the mother in creating this bond.

The contribution of our study is to have described and demonstrated the value of diverse midwifery practices in promoting the bonding process, based on mothers’ experiences. These findings may guide not only midwives in their practice, but also other professionals accompanying mothers and their partners within other (interdisciplinary) maternity care models beyond midwifery-led settings. Several elements mentioned by study participants appear relevant for healthcare professionals and agencies seeking to enhance person-centered and relational care, such as: Building a trusting relationship with mothers, being sensitive and responsive to their concerns, providing emotional support to new mothers throughout the process, promoting birth as a normal and positive experience, respecting mothers’ pace, and providing individualized interventions adapted to mothers’ specific needs. Study results also have implications for public health policies, such as emphasizing the importance of continuity of care across pregnancy, childbirth, and the postpartum period. Promoting continuity in relationships between healthcare providers and mothers and their partners, spanning the entire maternity process (from pregnancy to the postpartum period), fosters feelings of trust and security, both of which are crucial for women’s positive experiences of maternity and optimal maternal and neonatal health outcomes.

## Figures and Tables

**Table 1:** Examples of themes that emerged from the analysis: codes, units of meaning, and examples of verbatim quotes.

Theme: Giving Birth and Welcoming Your Baby at Your Own Pace (Timeframe: Labor and the First Moments After Birth)		
Code	Unit of Meaning	Examples of Verbatim
**0,03—Physical relationship with the baby**	**06_Cuddling, taking the time to look at the baby** Excerpts where women talk about taking the time to look at their baby, cuddling with them; eye contact and touch, sensations, closeness.	*You know, she was on me, and I was looking at her and I was just like, “She’s beautiful!” Seriously, she was beautiful. Well, she’s still beautiful. But you know, all parents say that about their babies. (…) It was really just a nice feeling of: “I’m happy with my baby.” And she was on me, you know, we stayed close together like that for a long time.* P20
**0,09—Intimacy and closeness with the baby**	**02_Cocoon allows for discovery, a family space** Excerpts in which women talk about a space of intimacy with their baby and/or partner that brings something concrete to the relationship.	*I held her close to me and my partner came into bed with me. The midwives were gone for a really long time. Then we slowly realized that the little girl was there.* P12 *I would say gentleness. In the sense of all the warmth and the little cocoon there. She was sleeping in bed, my partner lay on his back and she was lying next to him. We slept like that for a while at first.* P29 *You know, we stayed in our little cocoon there for a while. You know, staying in bed, watching her, sleeping, and feeding her.* P23
**0,14—Context of the first contact**	**02_Baby placed on the mother** Excerpts where women talk about how the baby was placed on them at birth.	*It was my partner who took the baby, actually, to take him out. Then he placed him on me. So that was really a beautiful moment.* P28 *The midwife said clearly “You can take her when you want…” I stayed on all fours for a moment, and someone said “Ah, it’s a girl!” I looked at my partner and then I said: “I’m ready to take her.”* P12
	**05_Situations related to the care context** Situations related to protocols and work organization that facilitate or interfere with contact with the baby.	*In any case, the midwives, everyone left at some point. They said: “Well, we’ll let you enjoy the moment.” (…) I felt like my midwife was watching over me.* P8 *You know, he was the one who held his head, and when he came out, he was in my boyfriend’s hands. So, I was like: “Wow!” It was a magical moment. (…) We went to the room. They gave us time alone together. The three of us were like glued together.* P26
	**09_Mother feeling in shape, feeling of well-being** Excerpts where women express how they feel.	*Well, of course it would change something in the sense that I was really happy to have control over my body afterwards, to be able to feel everything. I’m really proud of giving birth, and I think it’s a moment that’s truly “empowering”.* P29
**Theme: A bond with the baby during pregnancy** (Timeframe: Pregnancy)		
**0,20—Caregivers and care context**	**05_Provide confidence** Excerpts in which women talk about the feeling of confidence they have developed in relation to their midwife care, which extends beyond childbirth.	*The feeling grew stronger with each appointment with the midwives because they make it very human. You hear the heartbeat every time, they touch your belly and make you feel that there really is a baby. So, I think that at every appointment I felt something growing inside me.* P28 *Having a midwife, it’s not medicalized. I’m sure that has an effect on her. We feel confident, so the pregnancy is going well. So, your baby surely feels that.* P12
**0,15—Expectations and preparation for childbirth**	**04_Attitude, confidence** Excerpts where women talk about their attitude before giving birth.	*Throughout my pregnancy, as I said, I trust my baby and my body. I never felt any stress about giving birth. They know best what needs to be done.* P20

**Table 2:** Sociodemographic characteristics of participating mothers.

	Total (*n* = 10)
**Age** (average in years)	33 (29–37)
**Level of education** *n* (%)	
Secondary education	4 (40%)
Post-secondary education	6 (60%)
**Employment** *n* (%)	
Administration	3 (30%)
Healthcare sector	1 (10%)
Teaching	3 (30%)
Sales and services	1 (10%)
Natural resources, agriculture	2 (20%)
**Family structure** *n* (%)	
Two-parent	10 (100%)
**Family income ^a^** *n* (%)	
<49,999$	4 (40%)
50,000–79,999$	2 (20%)
80,000–99,999$	2 (20%)
>100,000$	2 (20%)

^a^ In 2022, the median family income (total income) for Quebec couples without children was CA $88,300 (Canadian Income Survey (2012–2022), adapted by the Institut de la statistique du Québec) [26].

**Table 3:** Pregnancy and childbirth data.

	Total (*n* = 10)
**Place of delivery** *n* (%)	
Hospital (HC)	1 (10%)
Birth center	7 (70%)
Home	2 (20%)
**Gestational age** (average in weeks)	40.4 (38.6–41.4)
**Duration of labor** (average in hours)	8.44 (3.77–12.65)
**Complications during labor or delivery** *n* (%)	3 (30%) ^a^
**Intervention on the baby following delivery** *n* (%)	2 (20%) ^b^
**Breastfeeding** *n* (%)	10 (100%)

^a^ 2 decelerations at the end of pushing, 1 tight circular, limp baby without spontaneous crying. ^b^ 1 bulb suction and 1 bulb suction + VPP 5 times (tight circular): APGAR: 4/9/10.

**Table 4:** Midwife support and its influence on bonding.

Main Themes	Definition	Related Midwives’ Actions
**Felt experience**	Physical, psychological, sensory, and emotional sensations experienced by the mother.	Offering the possibility of listening to the baby’s heartbeat, touching the belly, and feeling the baby’s movements. Talking to the baby and encouraging the mother to do the same.
**Rhythm**	Respecting the rhythms surrounding birth allows the mother to integrate her experience and positively promotes the establishment of her relationship with her baby.	Being in tune with each mother’s rhythm: allowing time for each woman’s subjective experience at every stage. Welcoming the baby at the mother’s pace: waiting until the mother is ready for the first physical contact and closeness, as well as for breastfeeding.
**Confidence and associated consequences**	Confidence allows the mother to feel more secure, less stressed, and more respected, increasing her overall sense of satisfaction and competence. It allows her to feel free to be herself and act according to her instincts, which positively influences her relationship with their baby.	Developing a trusting relationship with the mother. Listening actively and responding to the mother’s concerns. Informing the mother about the physiological process and the normal course of pregnancy. Promoting birth as a normal and positive experience. Providing emotional support to the mother and reinforcing her abilities.
**Environment**	An environment (regardless of the place of birth) that welcomes emotions and supports the transition to motherhood fosters the contact and bonding between the mother and her child.	Creating a favorable environment: calm, intimate, and safe, where the woman feels well accompanied and supported.
**Continuity**	Continuity of care during the pre-, peri-, and post-natal periods helps build a trusting relationship and enables the mother to focus on what she is experiencing and feeling and to connect with her baby.	Ensuring continuity through pre-, peri-, and post-natal follow-up. Adapting to the specific needs of the mother and her family.

## Data Availability

The datasets generated and/or analysed during the current study are not publicly available in order to maintain the confidentiality of participants, but are available from the corresponding author on reasonable request.

## Abbreviations

The following abbreviations are used in this manuscript:

RM1	Registered Midwife 1
RM2	Registered Midwife 2

## Appendix A. Interview Guides

### Appendix A.1. Interview Guide—Mothers

**Contextual information:**

The interviewer will review the sociodemographic questionnaire previously completed by the participant in order to have some background information about each participant’s situation.

**Main themes according to research objectives:**

Representations regarding the first contact with the newborn in relation to the context of childbirth:

- Representations of the mother in relation to the first moments spent with the baby (in general).
- Mother’s experience of pregnancy: specific details, expectations regarding childbirth and the first encounter with the baby.
- Childbirth experience: place, type of delivery, people present and their roles, sequence of events, specific details, interventions, procedures during labor and delivery, effects of the pandemic, institutional policies.
- Physical environment during birth (e.g., water birth, dim lighting or not).
- Links between the childbirth experience and the relationship established with the baby.
- First hours with the baby, memories (e.g., videos, photos, birth story)
- Factors that influenced the first moments.

Representations of the relationship with the baby during the first 2 postnatal months:

- Perceptions of her child and her relationship with her baby: first days, first weeks, first months (during the first 2 months).

### Appendix A.2. Interview Guide—Midwives

**Contextual information:**

Demographic data (including information on work experience) will be collected prior to the interview using an online questionnaire. The interviewer will review the information obtained prior to the interview in order to have some background information about each participant.

**Main themes according to the research objectives:**

*The interview is intended to complement the findings drawn from the analysis of the interviews conducted with the mothers, particularly regarding mothers’ experience with their baby and the factors that facilitate or interfere with that experience. Accordingly, the questions will be formulated in a way that incorporates comments previously reported by the mothers, in order to relate them to the midwives’ observations. For example: “Some of the mothers we met reported that […]. Is this something you have observed? Could you tell us about it?”*

- Observations regarding how the newborn is welcomed by the mother, by the parents.
- Links between the childbirth experience and the establishment of the relationship with the newborn.
- Elements that may interfere with or facilitate the establishment of the first contacts with the baby: environment, type of delivery, institutional policies, etc.
- Role of professionals in enhancing this experience during birth and the immediate postnatal period.
- Practices that concretely operationalize the establishment of the bond between the mother and her newborn.
- Conditions perceived as ideal for optimizing the establishment of the relationship with the newborn.
